# Turkey vultures tune their airspeed to changing air density

**DOI:** 10.1242/jeb.246828

**Published:** 2024-08-02

**Authors:** Jonathan A. Rader, Tyson L. Hedrick

**Affiliations:** Department of Biology, University of North Carolina at Chapel Hill, Chapel Hill, NC 27599, USA

**Keywords:** Air density, Equivalent airspeed, Gliding flight, 3D tracking, Flapping flight, Elevation gradient

## Abstract

Animals must tune their physical performance to changing environmental conditions, and the breadth of environmental tolerance may contribute to delineating the geographic range of a species. A common environmental challenge that flying animals face is the reduction of air density at high elevation and the reduction in the effectiveness of lift production that accompanies it. As a species, turkey vultures (*Cathartes aura*) inhabit a >3000 m elevation range, and fly considerably higher, necessitating that they accommodate for a 27% change in air density (0.890 to 1.227 kg m^−3^) through behavior, physiology or biomechanics. We predicted that birds flying at high elevation would maintain aerodynamic lift performance behaviorally via higher flight speeds, rather than increases in power output or local phenotypic adaptation. We used three-dimensional videography to track turkey vultures flying at three elevations, and data supported the hypothesized negative relationship between median airspeed and air density. Additionally, neither the ratio of horizontal speed to sinking speed nor flapping behavior varied with air density.

## Teaser

**Highlighted Article:** Turkey vultures respond to variation in air density across their geographic range by modifying their flight speed, thus maintaining similar glide angles without increasing energetic output or local morphological adaptation.

## INTRODUCTION

Life at high elevation, and the corresponding reduction of air density, presents a two-fold challenge to locomotor performance in flying animals (Altshuler and Dudley, 2006). First, a physiological challenge of low air density stems from the reduction of available oxygen for respiration (Altshuler and Dudley, 2006; Storz, 2007), which can lead to hypoxia and decreased metabolic power output. Second, reduced air density poses a physical challenge to fliers because, all else being equal (wing area, *S*; coefficient of lift, *C*_L_; and airspeed, *V*), lift forces (*F*_L_) change in proportion to air density (ρ) (Altshuler and Dudley, 2006; Dillon and Dudley, 2014):
(1)

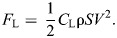
However, birds can be found at the highest elevations (>6000 m; Hiebl and Braunitzer, 1988; Liu et al., 2001; McCracken et al., 2009a,b; Tucker, 1968), suggesting that some species are able to compensate for these hardships. There are a variety of mechanisms that fliers can use to compensate for the aerodynamic consequences of flight in low air density. For example, high-elevation species tend to have larger wings relative to their body mass than their low-elevation counterparts (Feinsinger et al., 1979). Birds can also modulate their power output (Feinsinger et al., 1979), the proportion of flight time spent flapping relative to gliding (Schmaljohann and Liechti, 2009), or the amplitude of their wing strokes (Altshuler et al., 2004; Chai and Dudley, 1996) to compensate for changes in air density. Theoretically, birds could also modify flapping frequency, though this has not been observed (Altshuler and Dudley, 2006; Altshuler et al., 2004; Bruderer et al., 2010; Skandalis et al., 2017). One question that has received less attention, though, is whether birds compensate for reduced air density at high elevation behaviorally, such as through modulation of airspeed. Although not applicable to hovering flight or to landing and take-off, modulation of airspeed may otherwise offer a low to no cost method for maintaining flight performance across air density gradients.

Previous researchers have tracked individual birds (Sherub et al., 2016; Weimerskirch et al., 2000, 2003, 2016) and bird flocks (Alerstam et al., 1993, 2007; Green and Alerstam, 2000; Hedenström and Åkesson, 2016; Hedenström and Bone, 1993; Hedenström et al., 2002) flying across different altitudes using a variety of methods, including radar and GPS tracking. As predicted, birds have been shown to maintain higher airspeeds at higher altitudes (Green and Alerstam, 2000; Hedenström et al., 2002; Weimerskirch et al., 2016). For example, Himalayan vultures (*Gyps himalayensis*) increased their airspeed by 21% across an approximately 6500 m altitudinal gradient (Sherub et al., 2016), and birds in the spring migration had 12.8% higher airspeeds at 4000 m relative to birds flying at half that altitude (Bruderer, 1971). A question that remains underexplored is whether bird species with broad geographic ranges tune their flight speeds to environmental gradients across their range, or whether geographic heterogeneity is more likely to drive population-scale adaptation in morphology (i.e. wing size or shape; Altshuler et al., 2004; Bears et al., 2008; Skandalis et al., 2017).

To address this question, we studied three geographically separated groups of turkey vultures (*Cathartes aura*) at sites along an elevation gradient to determine whether different populations of these birds maintain different airspeeds in response to their local air density.

Turkey vultures are common throughout North America, inhabit an elevation range of >3000 m (del Hoyo et al., 1992) and have been reported flying at much higher altitude (Avery et al., 2011; Devault et al., 2005; Estrella, 1994). No available evidence suggests that any morphological differences in wing area or flight muscle mass exist among *C. aura* throughout their range, though this has not been addressed explicitly. However, they could alter their power output via increased flapping frequency or higher amplitude wing strokes when challenged by low air densities. Vultures are primarily gliding fliers (Arrington, 2003; Tucker, 1988, 1991, 1993), and could also adopt a strategy where they maintain the same true airspeed (which is the same as ground speed in still air) by increasing their glide angle, effectively increasing their power output by cashing in potential energy at a greater rate. Furthermore, *C. aura* consume almost exclusively carrion, which is a food resource that is sparsely distributed and highly ephemeral (DeVault et al., 2003; Kelly et al., 2007). Thus, it would seem advantageous for *C. aura* to minimize their energetic expenditure while foraging, suggesting that increased metabolic power output arising from modified flapping behavior is an unlikely adaptation to high elevation life.

We hypothesized that vultures accommodate flight at varying air densities by changing their true (i.e. observed) airspeed, increasing it as air density decreases. Mathematically, this is identical to the vultures maintaining the same equivalent airspeed (i.e. airspeed corrected for air density) at all air density conditions they experience. We further predicted that vultures would avoid activities that increase their energetic cost of flight: that they would not increase flapping in low air density, and that they would maintain similar glide angles throughout the elevation range – facilitated by the fact that drag is reduced in lower air density. This behavioral accommodation to reduced air density is not without consequence; the faster flight speeds increase the centripetal acceleration required for turning flight, making thermal soaring more challenging. Thus, although an increase in flight speed is perhaps the simplest accommodation to reduced air density, local morphological adaptation (such as relatively large wing area at higher elevations; Feinsinger et al., 1979) might better preserve the full range of vulture flight capabilities. In summary, we asked whether turkey vultures accommodate regional heterogeneity in air density behaviorally by modifying their flight characteristics (airspeed or flapping behavior) using data from vultures flying at three sites along a ∼2000 m elevation gradient.

## MATERIALS AND METHODS

### Vulture recordings

**Fig. 1.**
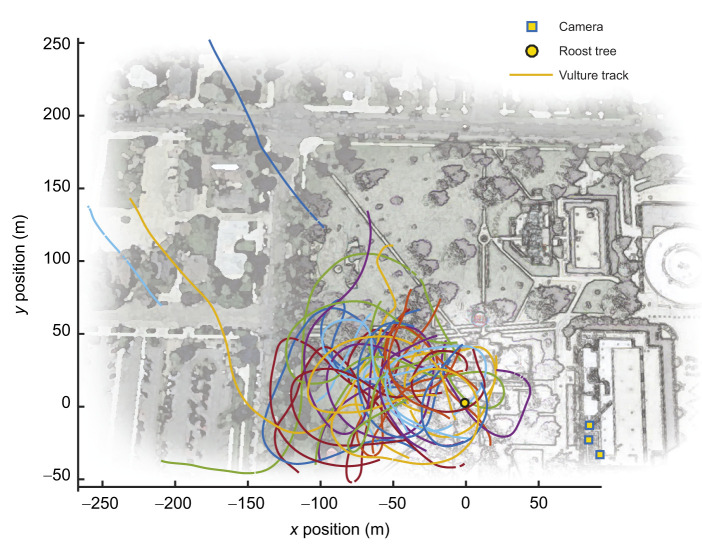
**Overhead view of the Laramie, WY, USA, recording site.** Blue and yellow squares denote camera locations atop the Biological Sciences building at the University of Wyoming, and the black and yellow circle shows the center of the roost trees. The multicolored tracks depict a sampling of the vulture tracks recorded from one recording bout.

We recorded turkey vultures [*Cathartes aura* (Linnaeus 1758)] returning to roost sites on 22 separate afternoons in May, June and July 2015 and September 2016 at three locations: the Orange County Landfill, Chapel Hill, NC, USA (35°58′9.23″N, 79°4′54.71″W), the University of Wyoming campus, Laramie, WY, USA (41°18′44.15″N, 105°35′1.04″W; see Fig. 1), and the Alcova Lakeside Marina, Alcova, WY, USA (42°31′45.99″N, 106°46′44.21″W). Each roost colony comprised >50 individuals.

Video data were collected with three digital SLR cameras (Canon EOS 6d, Canon Inc., Ōta, Tokyo, Japan) at 29.97 Hz, and images had dimensions of 1920×1080 pixels. The cameras were arranged in a staggered setup, with intersecting views of the tops of roost trees and airspace above and around them (Fig. 1). The distance between the cameras and the roost trees was approximately 65 m (at the Alcova site), 75 m (Laramie site) and 230 m (Chapel Hill site). Owing to the altitude at which the vultures approached the roost trees, and the requisite upward angle of the cameras, we were unable to use a standard wand calibration (e.g. Shelton et al., 2014; Theriault et al., 2014). Instead, we obtained a preliminary 3D calibration using optical information shared among cameras (i.e. the [*u*,*v*] pixel coordinates of objects seen in all cameras) digitized using the MATLAB (The MathWorks, Natick, MA, USA) package DLTdv5 (Hedrick, 2008), along with the pinhole-model characteristics of the cameras measured using custom Python routines (Jackson et al., 2016) in a sparse bundle adjustment calibration approach (Lourakis and Argyros, 2009). The initial calibration used static background points such as treetops visible in the camera images along with a few manually tracked vultures. After the initial calibration was complete, we automated tracking of the vultures using a computer vision workflow adapted from that used in Evangelista et al. (2017). In brief, birds were detected in each video file using a 30-frame moving average background subtraction routine plus a fixed background mask for the trees. The resulting background-subtracted images were cleaned with an erosion-dilation operation. The [*u*,*v*] pixel coordinate of each remaining foreground object was recorded as a possible vulture detection. These 2D detections from the three cameras were combined to compute a 3D point by searching the possible 2D point combinations for ones that produced a 3D reconstruction residual of less than 3 pixels. Points generated from either two or three cameras were accepted. Once the sets of 3D points for all video frames were generated, we joined the resulting 2D+3D datasets across time using a set of Kalman filters to predict the expected position of the birds from frame *n* in frame *n*+1 and then a Hungarian assignment operation to match the observations in frame *n*+1 to these predicted positions. Unmatched observations started new tracks, and tracks with more than 20 missed detections in sequence were discontinued. Tracks with fewer than 300 data points were dropped from the dataset. The calibration was refined using a second round of sparse bundle adjustment (Lourakis and Argyros, 2009) applied to the complete set of digitized points from the vulture tracks for each recording session. Three-dimensional camera calibrations based on bundle adjustment of intrinsic parameters (e.g. lens barrel distortion and pincushion) and extrinsic parameters (i.e. relative positions of the cameras and their orientation relative to horizontal) provide scale only up to a single unknown multiplicative factor and do not provide any information on the global orientation of the scene (Hartley and Zisserman, 2003). To add this information, we used the measured distances between the cameras to provide the scale factor and aligned the cameras to gravity using their onboard roll-leveling feature, and we measured their pitch inclination using a digital inclinometer affixed to the hot-shoe mount on the top of the base camera body. This two-pass calibration workflow improved calibration quality for points distant from the center of the scene. Finally, the scene was oriented to a geographic frame of reference by aligning to the compass vector between the base camera and the roost tree. Measurement precision in camera-based measurements necessarily declines with distance from the cameras (see Fig. S1 for quantification of dimensional uncertainty in the camera tracking in this study).

Individual bird 3D tracks in the scaled and aligned dataset were smoothed using a zero-lag digital Butterworth low-pass filter with an 8 Hz cut-off frequency. Velocity vectors were calculated from this smoothed position time-series by fitting a quintic spline polynomial and differentiating it. We added the wind speed vector (see below) to this ground reference frame velocity vector to obtain each bird's observed airspeed.

### Measuring vulture airspeed

First, vulture ground speed was calculated as the first derivative of vulture position with respect to time. Vulture airspeed was then obtained by subtracting the *x* and *y* components of the estimated wind speed (see below) from their respective ground speed components of the vulture tracks. No sustained thermal soaring behavior was observed during the recording periods, and we were unable to assess vertical movement of the air, so we assumed that non-flapping tracks represented gliding flight.

Wind and weather conditions during the recording sessions were recorded from the closest National Oceanic and Atmospheric Administration (NOAA) weather station for all locations, and from a rooftop-mounted weather station atop the University of Wyoming Biological Sciences building, adjacent to the roost. Because of the distance between the recording sites and the weather stations, and because ground-level wind conditions may not reflect the conditions experienced by the birds, we also estimated the magnitude and direction of wind conditions during each recording session from the ground speeds of the birds as they flew in different directions, following the ‘circle wind’ method of Sherub et al. (2016). To a first approximation, absent of any wind or other directional factors, bird ground speeds are not expected to vary with flight direction such that a plot of the two components of their horizontal velocity vector form a circle centered on [0,0]. A wind alters the center of the circle. For example, a 5 m s^−1^ wind in the +*x* direction moves the center to [5,0] (see Fig. S2 for an example using previously published chimney swift flight track data; Evangelista et al., 2017). Thus, we estimated the wind speed and direction experienced by the vultures as the center point of a circle fitted to the *x* and *y* components of their measured groundspeed vectors over a recording session. This method depends on having a sample of flights headed in all compass directions, even though individual flights themselves need not be circular. Consequently, we omitted trials with vulture tracks comprising less than 90% of the full circle (see Fig. S3 for sample data from a single vulture flight recording bout).

The ‘circle wind’ method applied as described above assumes that birds do not alter their flight behavior with wind. This is likely a poor assumption for birds in directional flight, as was the case for our vultures, so we extended the analysis described above to account for birds altering their airspeed to optimize cost of transport with a tail, head or cross-wind (details provided below). Further, we used a linear regression to check the agreement between the ‘circle wind’ estimates and the recorded windspeeds from the weather stations using the ‘lm’ function in R (stats library, https://www.r-project.org/). The weather station data generally agreed with our ‘circle wind’ fit (*F*_1,27_=7.93, *P*=0.009), but we attribute the moderate fit (adjusted *r^2^*=0.20) to the distance between the weather stations and our sites, coupled with regional variation and topographic influence in wind speed.

### Adjusting the ‘circle wind’ measurement method for maximum range flight behavior

The ‘circle wind’ method uses the ground speed of birds in flight to determine the prevailing wind direction. In its most simple form, the analysis assumes that birds use the same airspeed regardless of heading, and uses the flight speed at different headings to determine the origin of the circle revealed by a plot of the [*V_x_*,*V_y_*] velocity components in the horizontal plane. As shown in Fig. S2 for data from the chimney swift roosting flock described by Evangelista et al. (2017), and for vulture data on a low-wind day (Fig. S3), these results often show little deviation from circularity. However, that result is only expected when birds do not adjust airspeed in response to wind direction. Although this might be the case for the swifts, which have already reached their destination (the chimney) and are simply remaining in the air above until their sunset roosting time, birds flying toward a destination are expected and have been shown to adjust airspeed in response to wind speed to minimize cost of transport (e.g. Spear and Ainley, 1997).

This alternate assumption, that birds alter airspeed to minimize cost of transport, produces a distorted circle, as shown in simulated data (Fig. S4) from a numerical solution for a black vulture flying over a full circle of headings, minimizing transport according to the glide polar from Parrott (1970) and a wind at [4.95, 4.95]. In this case, fitting a circle to the simulated data produces a wind speed estimate of [3.82, 3.82], approximately 77% of the actual value (Fig. S4A). This ratio persists for a wide range of wind speeds and remains highly linear (Fig. S4B) for wind speeds of up to half of the maximum glide distance speed for the glide polar used for the analysis. Furthermore, the ratio is not strongly sensitive to the details of the glide polar; using an alternate glide polar derived from the data recorded here and assuming the animals do not tune airspeed to wind speed increased the slope of the relationship in Fig. S4 by only 8%. Thus, the investigation of how the circle-fit method for determining wind speed and direction interacts with wind-aware flight behavior by birds minimizing cost of transport reveals that the circle-fit results require a simple multiplicative correction of 1.29. We applied this correction to the vulture airspeeds prior to subsequent analysis. We also tested alternate assumptions, specifically assuming no flight behavioral response to wind (i.e. a multiplicative factor of 1.0) or assuming a glide polar with a lower minimum power speed (10.3 versus 13 m s^−1^) and producing a correction factor of 1.39 instead of 1.29.

### Air density and its relationship with vulture airspeed

Air density (ρ) was calculated from mean barometric pressure, ambient air temperature (*T*) and dew point readings from the NOAA weather stations during the recording periods using the formula for density of moist air from Brutsaert (2013, p. 37):
(2)

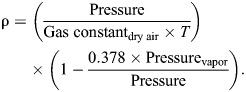
We calculated median airspeeds for each track, and from those the median airspeed of vultures during the entire recording session. We were unable to identify and track identities of individual birds, and birds readily transited into and out of the camera field of view multiple times during recording bouts, so it is highly probable that individual vultures contributed multiple tracks to the dataset by appearing during successive recording sessions at the same site. However, these individual replicates would have been collected at separate points in time and thus represent flight under different conditions (e.g. air density, wind direction relative to flight direction, and even body mass). Nonetheless, we took multiple steps to avoid any biasing effects that such pseudoreplication might impose. We first conducted our analyses in a mixed-effects framework with track ID and recording session as random effects. We also tested multiple schemes of collapsing the data to median values for (1) each track and (2) each recording session. There were multiple recordings per day; however, we treated each of these separately because ambient temperature and humidity change throughout the day, so air density also varied among recordings. Subsequent analyses of the effects of air density and wind conditions upon airspeed were conducted on those medians, and we compared the results to those produced by our mixed-effects model and those produced by analyzing the full, non-collapsed dataset.

We hypothesized that vulture airspeed would decrease in a non-linear fashion as a function of increasing air density (ρ), which we investigated using a pair of regression models. First, we fit a mixed-effects model to the entire dataset of vulture tracks with ρ and the ‘circle wind’ estimate of wind speed as the fixed effects, and with the track unique identifier and recording session as random effects using the ‘lmer’ function in the lme4 package (https://CRAN.R-project.org/package=lme4) in R. In doing so, we were able to simultaneously model the effects of air density and wind speed on vulture flight speed, while also controlling for changing environmental conditions and our inability to conclusively maintain individual identifications of the birds while they were out of the camera views.

We also fit a quadratic regression model to evaluate the relationship between median airspeed and ρ. All regression models were implemented using the ‘lm’ function in R (stats package; https://www.r-project.org/). Second, we also used a linear approximation of the theoretical relationship between airspeed and ρ and the median airspeed of vultures at the maximum ρ in the sample (ρ=1.227):
(3)

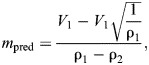
where *m*_pred_ is the predicted linear approximation slope, ρ_1_ is the highest air density in the sample, ρ_2_ is the lowest air density in the sample, and *V*_1_ is the median airspeed of vultures flying in the highest ρ conditions in the sample. The estimate of airspeed at ρ_2_­ is based on eqn 5 in Pennycuick (2001). Although the physical relationship between *V* and ρ is non-linear, using this linear approximation allowed us to generate a predicted slope for the relationship between median airspeed and ρ that we could evaluate in a hypothesis-testing framework. Based on this framework, we predicted a slope of −5.47 m^4^ kg^−1^ s^−1^. We used a series of Wald χ^2^ tests to assess whether any of the model slopes differed from *m*_pred_ or from each other using the ‘linearHypothesis’ function in the car package (Fox and Weisberg, 2019) in R.

We further used a set of multiple linear regression models to test the impact of wind speed on the relationship between *V* and ρ. We used the corrected Akaike's information criterion (AICc) to select the best fit among these candidate models. Finally, to test whether our results were sensitive to the particular assemblage of tracks in our sample, we randomly subsampled 50% of our track data (with replacement), fitted both the quadratic and linear approximation models to the subsampled median vulture airspeed (*V*_med_) and ρ, and bootstrapped this 10,000 times (see Fig. S5). To check whether vultures were maintaining similar glide angle across the range of air densities, we calculated glide angle for the vultures as:
(4)

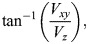
where *V_xy_* is the horizontal airspeed and *V_z_* is vertical airspeed, and used an ordinary least squares (OLS) regression to test for a relationship between glide angle and ρ.

### Air density and flapping behavior

The automatic tracking algorithm detects and tracks the visual centroid of the vultures in each video frame (as opposed to a fixed point on the body, such as the head). Because of this, and owing to the large size of the birds' wings, their tracks appear as a sinusoidal wave pattern when the birds flap, contrasting with comparatively smooth gliding tracks. We exploited this to assess whether the vultures flapped more in lower air density, a sign that they might be compensating for decreased lift by modulating power output. We used a custom MATLAB program to detect that characteristic sinusoidal track pattern and coded each video frame of each track with a binary (0) gliding or (1) flapping. We then used the mean to quantify the proportion of the track the bird spent flapping versus gliding. Further analyses of flapping behavior were restricted to tracks closer than 350 m from the cameras, as this appeared to be the maximum distance at which flapping is detectable (see Fig. 3 and Fig. S1). We also excluded tracks within 50 m of the roost to avoid tracks in which the birds were making their final landing approach, which was characterized by a large amount of flapping not necessarily related to air density. Data were again collapsed to medians for each recording bout. We used OLS regression to look for a relationship between incidence of flapping and ρ. Finally, we assessed whether the proportion of flapping in tracks using OLS regression on the mean or the probability of detecting flapping (binary logistic regression) varied with wind speed.

## RESULTS

### Vulture tracks, air density and flight speeds

**Fig. 2.**
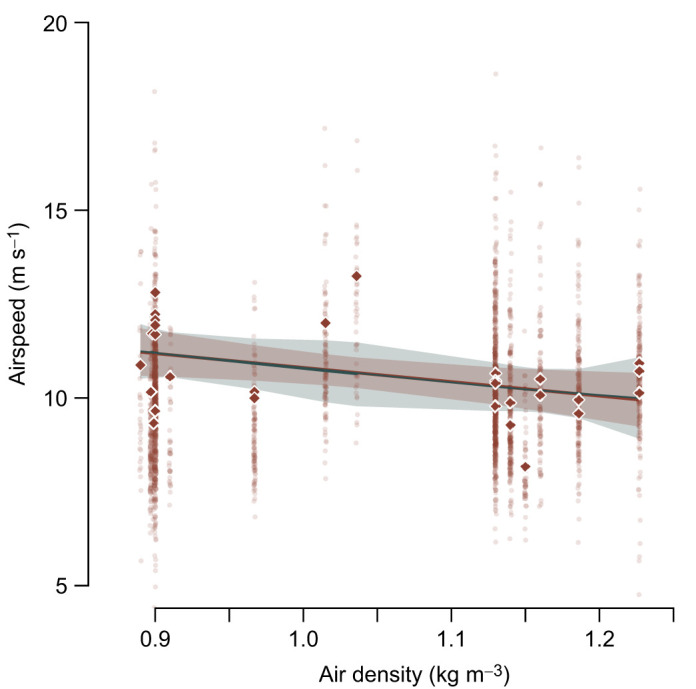
**Median airspeed decreased with increasing air density.** Transparent data points show median values for each track, highlighting the large variation in the sample. Quadratic and linear regression analyses were conducted on median values for each recording session, depicted by solid diamonds. The green line shows the quadratic regression, and the gray shaded region is its 95% confidence interval. The brown line (partially obscured by the overlapping green quadratic regression) shows the linear regression with its 95% confidence interval (brown shaded region). A linear mixed-effects model applied to the full, un-collapsed data but with random effects for recording session and individual flight track yielded a substantially similar estimate of the slope. For the sake of clarity, those results are not included here (see Table 1).

We collected 2458 vulture flight tracks representing 18 h of vulture flight time. Mean reprojection error, a measure of the accuracy of the 3D reconstruction from the three 2D camera images, was 0.61 pixels. Note that for calibrations with similar dimensional metric error, the pixel reprojection error will decline with subject distance. Thus, the associated metric position uncertainty varies with bird distance from the cameras; at 350 m, uncertainty was 0.51 m, with the majority in the camera viewing axis (Fig. S1). Median vulture airspeeds, summarized by recording bout, ranged from 8.17 to 13.24 m s^−1^ (see Table S1) with an overall median airspeed of 10.45±0.87 m s^−1^. There was a large amount of variation in airspeed among vulture flight tracks in each recording session; median absolute deviations ranged from 14% to 28% of the median (Fig. 2). After correcting for ambient temperature and relative humidity, ρ ranged from 0.890 to 1.227 kg m^−3^ (see Fig. 2).

**Fig. 3.**
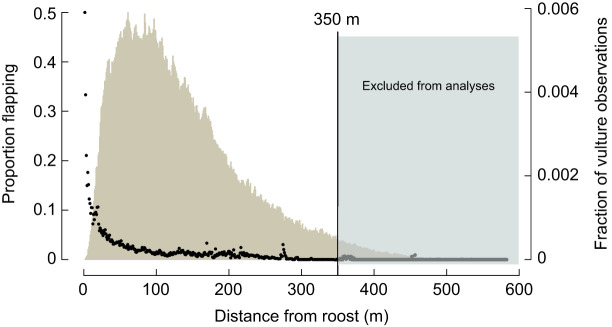
**Proportion of detected flapping events, indicated by black points, decreased with distance from the roost trees (left *y*-axis).** The histogram depicts distribution of tracked birds, relative to the roost position (right *y*-axis). The ability to detect flapping diminished with distance from the cameras, so tracks greater than 350 m from the roost were excluded from further analyses.

**Table 1.**
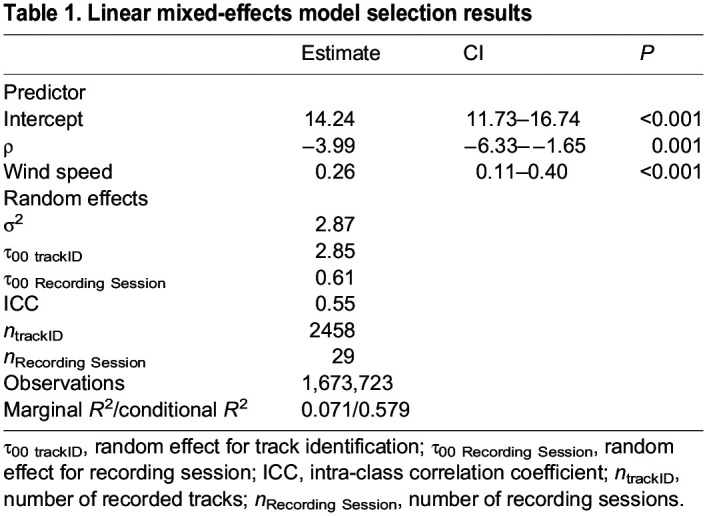
Linear mixed-effects model selection results

A linear mixed-effects model that included the recording session and individual flight track identifiers as random effects found highly significant coefficients for both fixed effects: ρ and circle windspeed (see Table 1). The estimated value for ρ from this model was −3.99 (standard error=1.20), and the estimate for wind speed was 0.26±0.07 (*P*=0.001). We also fit a quadratic regression model to *V*_med_ and ρ, as this relationship is known to be non-linear. Perhaps owing to the large variance in vulture flight speeds during the recording sessions or to the small range of air density that we sampled, we found that the non-linear term in the model was not statistically significant (*P*=0.91) and that the regression converged with the result of the linear approximation (see Fig. 2). Indeed, all of the linear models of *V*_med_ versus ρ were favored by AICc (Table 2). We stress that our result does not imply that the relationship is in fact linear, merely that our data are not sufficient, or are sampled over too narrow of a range of ρ to find statistical support for the non-linear component of the known physical relationship. Our results were robust to different sampling approaches; the regression coefficients that we recovered from our quadratic and linear approximation models did not differ from the median values of the coefficients in our bootstrap test (see Fig. S5).

**Table 2.**
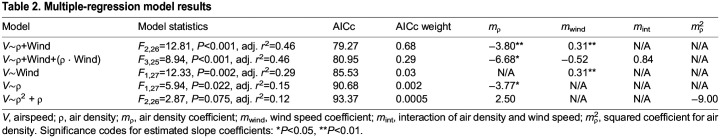
Multiple-regression model results

The best performing multiple regression model, via AICc, included effects of ρ and wind speed (AICc weight=0.68). *V*_med_ decreased with ρ (estimated slope *m*_ρ_=−3.79, *F*_2,26_=12.86, slope *P*=0.005; see Fig. 2) and increased with the estimated wind speed (*m*_wind_=0.31, slope *P*<0.001). Full results for the linear models are presented in Table 2. The estimated slope of the relationship between *V*_med_ and ρ was not statistically distinguishable from the predicted slope (*m*_pred_, Eqn 3) in the best performing model, which included both ρ and wind speed (Wald χ^2^ test, *F*_1_=2.54, *P*=0.12), the mixed-effects model (Wald χ^2^ test, *F*_1_=0.13, *P*=0.72) or the model that included only ρ (Wald χ^2^ test, *F*_1_=0.05, *P*=0.82). In our estimates of wind speed, we applied a small multiplicative correction factor (1.29) to the vulture airspeeds to account for the birds adjusting their behavior in response to wind speed and direction (see Materials and Methods). We tested whether this influenced our slope estimate in the *V*_med_ versus ρ relationship by iterating the top-performing multiple regression model (which included both ρ and wind speed) on the corrected and uncorrected airspeeds, and found that the slopes were statistically indistinguishable (Wald χ^2^ test, *F*_1_=0.013, *P*=0.91). We also found a positive relationship between *V*_med_ and wind speed using a linear regression (*F*_1,27_=12.33, *P*=0.002). Finally, there was no relationship between vulture glide angle and ρ (*F*_1,10_=2.045, *P*=0.18; Fig. 5).

### Flapping analysis

**Fig. 4.**
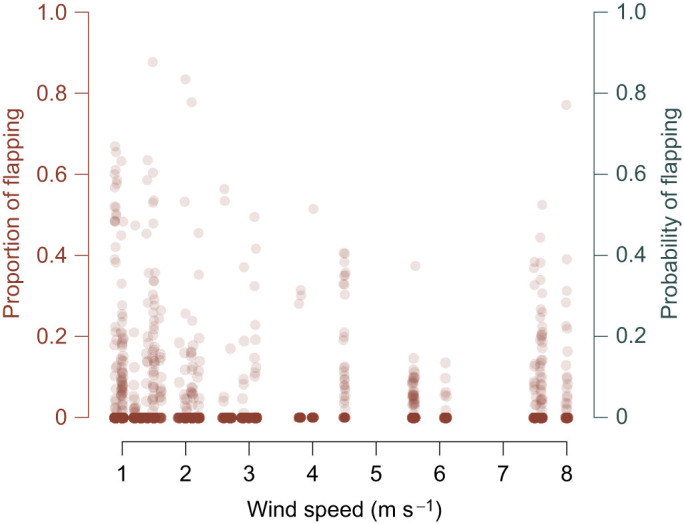
**Flapping behavior as a function of wind speed.** The proportion of the tracks where flapping was detected (brown circles, left *y*-axis) was low across all wind conditions but increased slightly (though not significantly) with wind speed. Points show individual track values to depict the dispersion of the data and are depicted with transparency to show overlapping points – dark brown points show high overlap. Analyses were conducted on median values for each recording session.

We were only able to reliably detect flapping in tracks that were in close proximity to the cameras, so we restricted analysis of flapping behavior to tracks that were within 350 m of the roost, and at least 50 m away from it to avoid analyzing landing tracks. Fortunately, 84% of the tracks in the overall dataset fit these criteria (see Fig. 3). There was no relationship between the proportion of flapping in the tracks and ρ (OLS regression, *F*_1,27_=2.14, *P*=0.15); however, the proportion of flapping in tracks decreased steeply away from the roost (Fig. 3). There was also no relationship between wind speed and the proportion of tracks where flapping was detected (*P*=0.72; see Fig. 4).

**Fig. 5.**
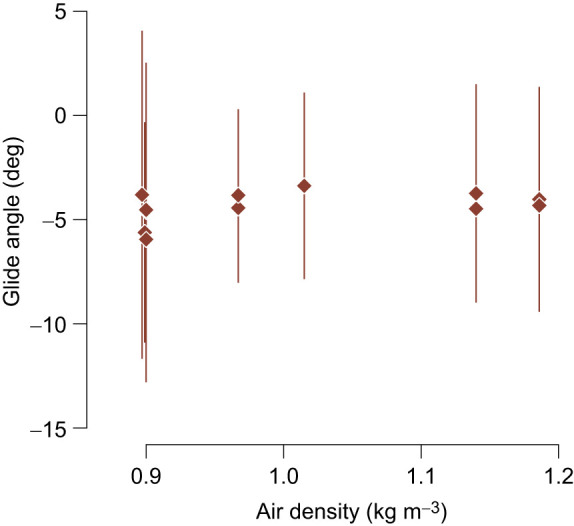
**There was no relationship between vulture glide angle and ρ.** Brown diamonds depict median glide angle for each recording day and error bars show ±1 median absolute deviation. We tested for a relationship in these data using ordinary least-squares regression, but there was no significant slope (*F*_1,10_=2.045, *P*=0.18).

## DISCUSSION

### Summary of results

We predicted that median airspeed in vultures flying across a range of elevations and ambient conditions would increase in response to decreasing air density (ρ). Based on a simple linear approximation of the relationship between airspeed and ρ, we predicted a slope of −5.47 m^4^ kg^−1^ s^−1^ across the sampled range of ρ. We found that median vulture airspeed (*V*_med_) largely conformed with this prediction (estimated slope=−3.99, confidence interval=−6.33 to −1.65), despite a large amount of variation among individual tracks. Furthermore, we found that vultures do not modify their flapping behavior in response to air density or wind conditions. These results agree with prior observations of Himalayan vultures (*Gyps himalayensis*) and migrating birds tracked via GPS (Bruderer, 1971; Sherub et al., 2016). We show here that mechanisms by which individual birds compensate for low air density as they climb to higher altitudes also manifest among populations of birds residing along elevation gradients. The ability to behaviorally accommodate environmental gradients may mitigate selective pressures that would otherwise drive local phenotypic adaptation (Huey et al., 2003; Muñoz, 2022).

### Increased airspeed compensates for decreased air density

Drag forces also decrease with air density (Vogel, 1981), reducing a glider's resistance to gravitational acceleration, which is a likely mechanism for the observed increase in airspeed. However, this assumes that birds among the different populations are geometrically similar, having roughly the same wing loading and wing shape, and that morphological disparity (the variation in body shape and size) is roughly equivalent across populations. Local adaptation of wing morphology to high elevation in the sample would likely have manifested in less change in *V*_med_ relative to ρ, or a difference in the ratio of sinking speed (*V_z_*) relative to horizontal speed (*V_xy_*). There was no difference in glide angle across the sampled range of air density (Fig. 5). This, plus the agreement between the predicted and estimated slopes of the *V*_med_ versus ρ relationship, suggests no localized adaptation in wing morphology or loading. Further, if the increase in airspeed is simply a passive effect related to the reduction of drag, it implies that birds do not alter their flapping behavior (Schmaljohann and Liechti, 2009) or increase their glide angle in low density air. Vultures in the recordings examined here flapped more as they neared their roost trees, perhaps as part of their approach and landing maneuvers. However, neither the probability of birds flapping nor the proportion of time they spent flapping increased in response to either ρ or increased wind speed. Taken together, these results do not indicate any localized behavioral or morphological adaptations to maintain similar airspeed among populations of turkey vultures residing at different elevations.

Our sample sites, which encompass a 27% reduction in air density, captured the variation of flight conditions that vultures experience during typical flight bouts across their geographic range, but may not reflect extreme conditions that vultures sometimes experience. Vultures have been recorded at altitudes exceeding 1000 m (Avery et al., 2011; Treep et al., 2016), and anecdotal reports suggest that their maximum flight altitudes may be much higher (up to perhaps 6000 m). Air density decreases exponentially as altitude increases, necessitating disproportionally greater airspeed increases to maintain lift as birds climb higher, so it is possible that the relationship that we have described between *V*_med_ and ρ might change for vultures flying at especially high altitudes, requiring that they modify their flapping behavior or glide angle. Despite some observations of extremely high vulture flight, tracking data suggest that typical vulture flight altitudes are much lower, around 150 m above ground level (Avery et al., 2011; Devault et al., 2005), well within our sampled elevation range.

Our study is not without caveats. First, our measurement of air density is based on recordings of ambient temperature and relative humidity taken from the nearest weather station operated by NOAA. However, our recordings were not directly adjacent to the NOAA stations, meaning that the ambient conditions at our precise study locations may have differed slightly from the measured conditions. Furthermore, the NOAA recordings were taken at approximately 1 h intervals, whereas each of our recording sessions lasted only 15 min. We matched each recording session with the best temporal match from the NOAA data, but were unable to account for short-term fluctuations in ambient conditions. We do not expect that temperature and relative humidity rarely varied by amounts that would affect our results over the restricted geographic distances between the NOAA stations and our recording sites or the time scales of our recordings, and therefore find it unlikely to have systematically biased our findings. Finally, in addition to the geographic and temporal averaging of our density measurements, we were similarly unable to account for differences in air density experienced by vultures flying at different altitudes above the cameras. The range of vulture altitudes we recorded was on the order of tens of meters, and the variation in air density across the altitude range of the tracks would have been minimal relative to the elevation range of our study and would have been further attenuated by any local air currents.

The issue of pseudoreplication is a complicated one in studies such as this, arising from multiple sources. Therefore, we adopted a pluralistic approach to the analysis, conducting our tests of the relationship between air density and airspeed in a variety of ways, with varying assumptions. We conducted the analysis with the entire dataset, with the data collapsed to medians of individual tracks, and with the data collapsed to medians for each recording bout. We emphasize that all approaches yielded similar results. That is, the slopes estimated by each of these approaches were all indistinguishable from our predicted slope, via a series of Wald tests. For both the track and recording bout summaries, the resulting slopes are statistically indistinguishable from the hypothesized slope and from each other. The large power of the non-collapsed dataset (>1.6 million observations at the camera frame rate of 1/30th per second) does allow us to find a statistical difference between it and the other slopes; however, when we conduct a Wald test with that slope as the frame of reference, the test does not yield significant results. We also conducted a linear mixed-effects model on the full dataset with air density and circle wind speed as the fixed effects and track ID and recording session as random effects, and the slope estimated by this model also conforms to the rest of the analyses. We favor this mixed-effects approach and our original approach of collapsing the data to medians for each recording session because these control for the pseudoreplication we know to be present. We also find it comforting that different schemes of collapsing the data (or not) do not bias the outcome, and we therefore suggest that our multifarious approach here could be valuable to future studies with similar unaccountable replication.

### Wind effects

Wind conditions on our sample days varied from calm to heavy, gusty winds. The best performing model included both air density and wind speed as predictors for vulture airspeed. Our estimate of wind speed is based upon its influence on the measured groundspeeds of the birds flying at different angles relative to the wind, adjusted for expected flight behavior for optimal transport (see Materials and Methods and Figs S2–S4). Birds are known to increase their airspeed when faced with a headwind (Bloch and Bruderer, 1982; Spear and Ainley, 1997), though the magnitude of the increase varies among birds that differ in their flapping behavior (Spear and Ainley, 1997; Weimerskirch et al., 2000). Further, birds tend to decrease airspeed when flying with a tailwind, albeit this reduction varies among taxa, and this reduction is lower in magnitude than the amount birds increase their airspeed in headwinds (Bloch and Bruderer, 1982). Together, the increased airspeed in headwinds and slightly decreased airspeed in tailwinds leads to an overall trend of higher airspeeds during windy conditions. Indeed, we found a positive relationship between the estimated wind speed and median airspeed of the vultures. We interpret this result as vultures modifying their flight behavior to maintain forward progress toward their destination in the face of strong headwinds. The wind speed during the windiest recording sessions was nearly equal to the minimum power speed and thus sufficient to noticeably influence bird airspeed (Pennycuick, 1989). Despite the wind effect, the relationship between vulture airspeed and air density is statistically indistinguishable from the slope that we predicted based on the elevation and ambient temperature and humidity at our sample sites. Therefore, though the vultures clearly reacted to windy conditions, air density was still the dominant influence on their flight speed.

### Behavioral accommodation can limit morphological evolution

Behavior is classically believed to be a driver of evolutionary divergence: the adoption of preferences for novel resources and conditions exposes species to selective pressures that foster adaptive evolution and potentially drive speciation (Blomberg et al., 2003; Gómez-Bahamón et al., 2020; Mayr, 1960; West-Eberhard, 1989). Conversely, behavior can also have an inhibitory influence on adaptive divergence in other traits, a phenomenon known as the Bogert effect (Huey et al., 2003; Muñoz, 2022). The Bogert effect has been explored largely in the field of comparative physiology (e.g. Farallo et al., 2018; Huey et al., 2003; Marais and Chown, 2008; Muñoz and Losos, 2018). For example, among some squamate and salamander lineages, the ability to behaviorally thermoregulate has inhibited evolution of physiological tolerance to new regions with novel thermal regimes (Farallo et al., 2018; Muñoz, 2022; Muñoz and Bodensteiner, 2019). Similarly, if animals are capable of behaviorally accommodating variation in environmental features that influence their locomotor performance (e.g. air density), the pressure for local morphological adaptation may be mitigated. As such, explicit studies of the Bogert effect have the potential to be informative in the fields of functional morphology and comparative biomechanics, as well (Muñoz, 2022). Our results suggest that the type of behavioral accommodation that is the hallmark of the Bogert effect might be at play among turkey vultures; however, future surveys of flight-related morphology among vulture populations would reveal whether and to what degree local morphological adaptation is also present.

### Concluding remarks

Animals interact with their physical environment to move, forage, migrate and a host of other functions, and their ability to do so effectively can be limited by physical constraints imposed by their environment. We showed that turkey vultures respond to a fundamental environmental gradient that could impact their flight performance, the decrease of air density at high elevation by increasing their flight speed. The tool that we used was developed for field studies of biomechanics (Hedrick, 2008; Hedrick et al., 2018; Shelton et al., 2014; Theriault et al., 2014), but this study also demonstrates that tools from the biomechanics toolchest can be successfully applied to ecological questions.

## Supplementary Material

10.1242/jexbio.246828_sup1 Supplementary information
