# Supplementary material for: Turkey vultures tune their airspeed to changing air density
Source: J Exp Biol. 2024 Aug 2;227(15):jeb246828. doi: 10.1242/jeb.246828 (PMC11418199; doi:10.1242/jeb.246828)
Supplement: Supplementary information [file jexbio-227-246828-s1.pdf]

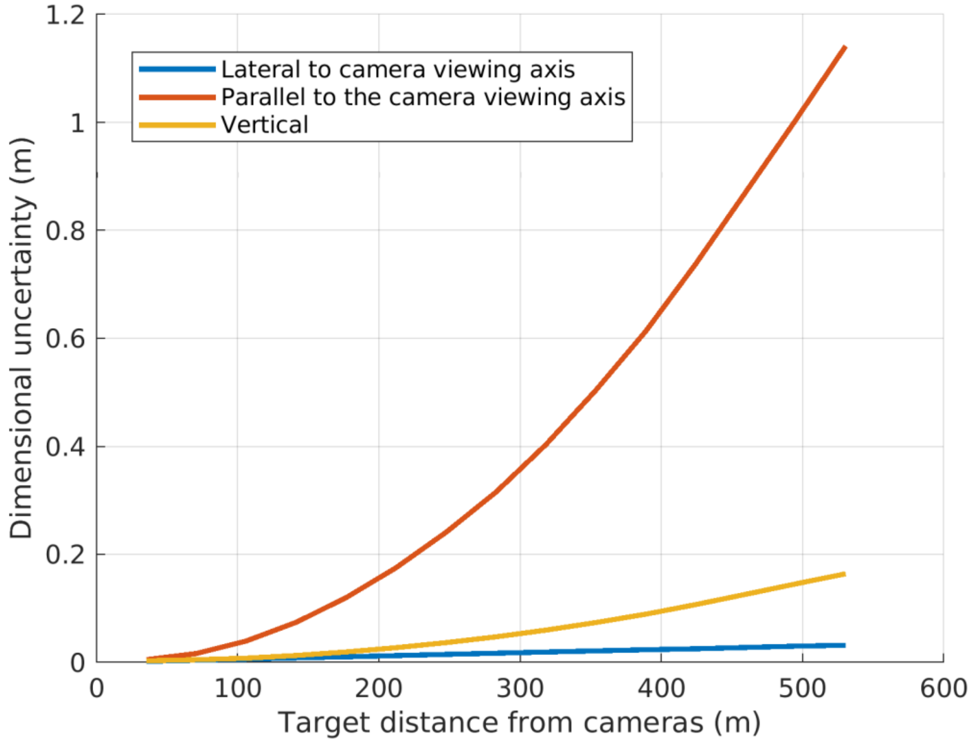

Fig. S1. The dimensional uncertainty associated with the mean reprojection error (0.61 pixels) for the vulture flight recordings. Dimensional uncertainty varies with distance from the cameras and is greatest in the direction parallel to the camera viewing axis. Note that tracks beyond 350 m from the cameras were excluded from our analysis because of the difficulty of identifying flapping from the vertical oscillations of the vulture images at greater distances.

Journal of Experimental Biology • Supplementary information

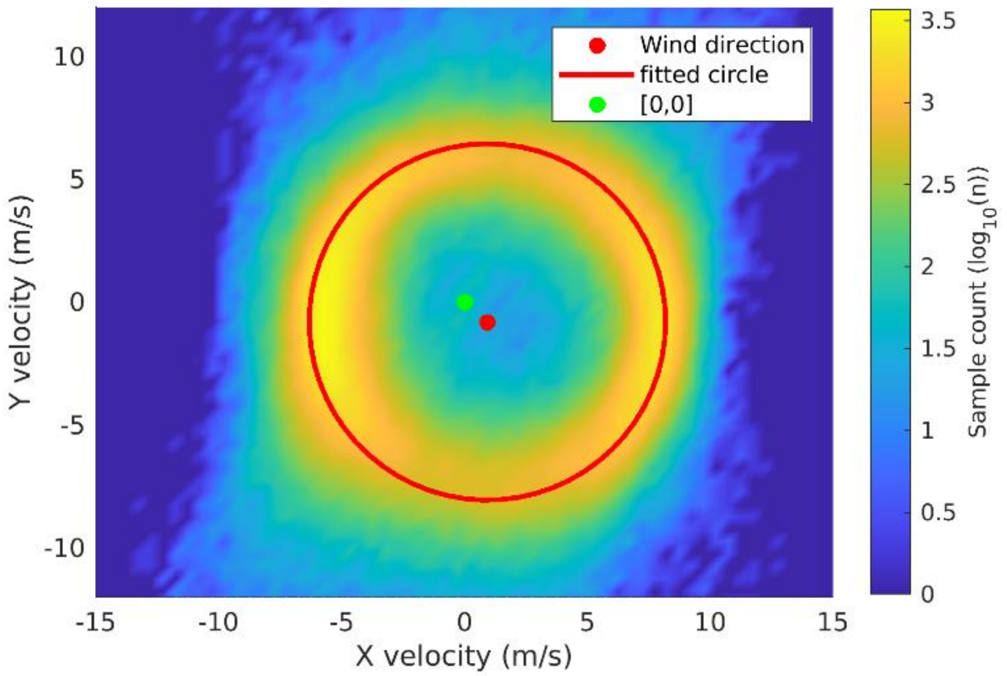

Fig. S2. The circle wind method applied to Chimney Swifts loitering above a roost.

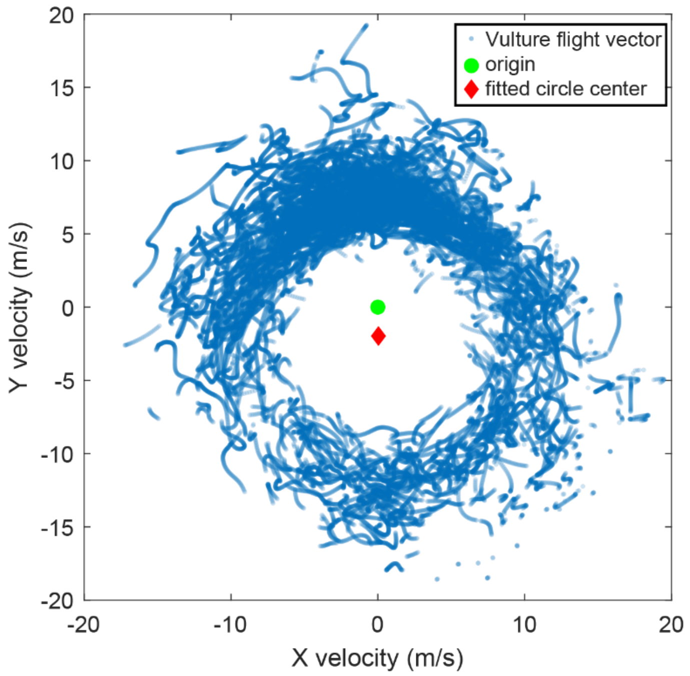

Fig. S3. Horizontal flight vector data from one of the vulture recording bouts in this study. While less densely sampled than the chimney swifts, the same circular pattern is apparent. Vulture flight vector samples are opaque such that more densely sampled regions are darker.

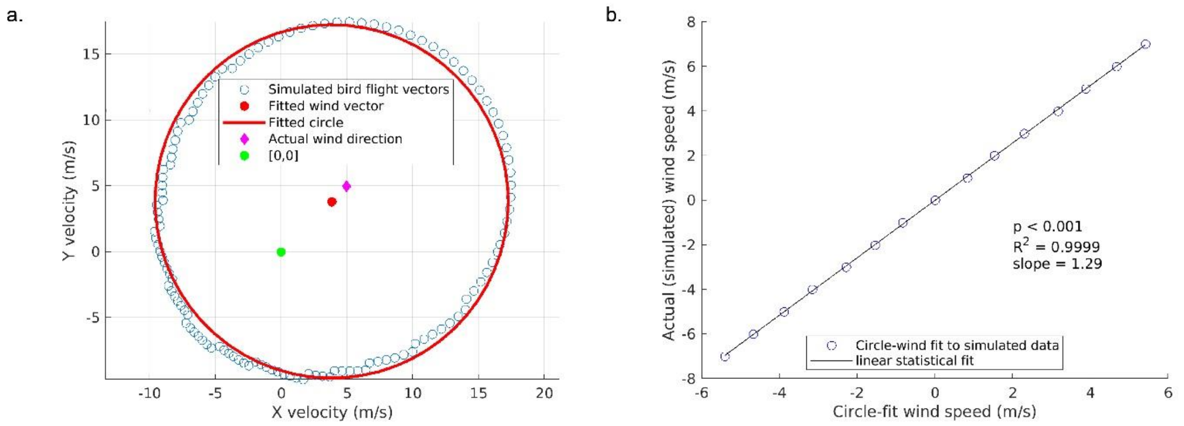

Fig. S4. a. The circle wind method applied to simulated vulture data assuming minimum cost of transport in a 7 m/s wind and using a Black vulture glide polar measured experimentally using a wind tunnel (Parrott, 1970). Note that discrete points in the glide polar measurement produced jumps in the simulated vulture flight vector. b. The relationship between the simulated actual wind speed and the circle-fit wind speed estimate calculated from a simulated vulture optimizing cost of transport.

Journal of Experimental Biology • Supplementary information

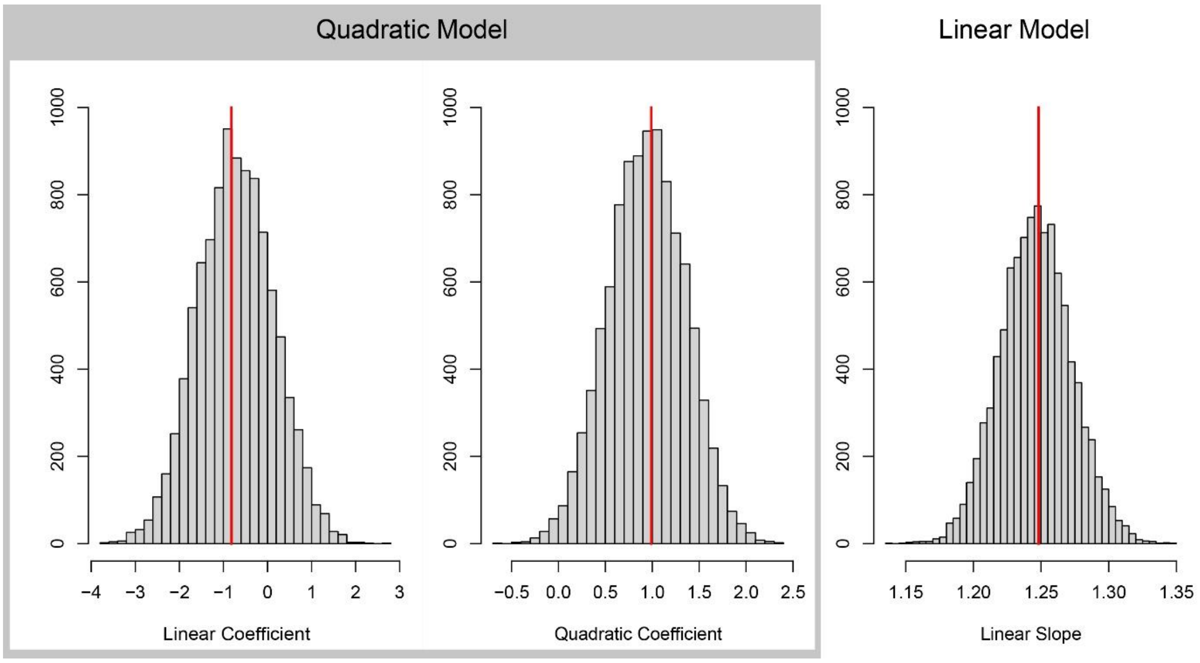

Fig. S5. We subsampled 50% of the track data (with replacement), recalculated the regression coefficients for both the quadratic and linear fits. We bootstrapped this 10,000 times and plotted histograms depicting the distributions of the coefficients. The red vertical lines denote the values of the coefficients recovered from analysis of the full dataset.

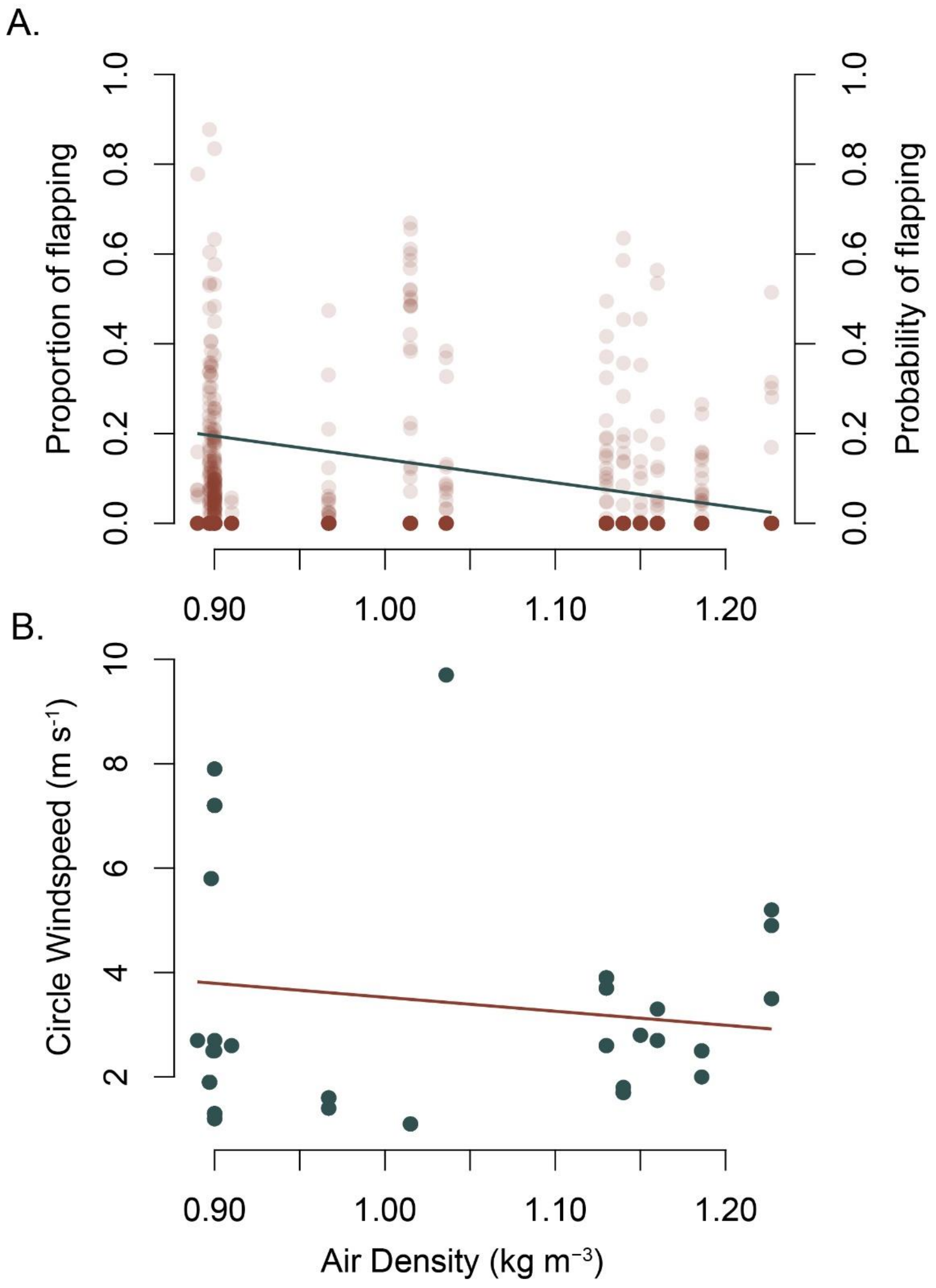

Fig. S6. A) Shows a negative relationship between the probability of detecting flapping and the proportion of flapping observed in vulture tracks across the sampled range of air density. B) The low air density localities were also the windiest recording sessions, leading to a negative trend of wind speed across the gradient of air density.

Journal of Experimental Biology • Supplementary information

Table S1. Summary of vulture tracking results.

Data ID	Location	Air Density (kg/m^3)	Median Equivalent Airspeed (m/s)	Median Sinking Speed (m/s)	Percent of Flapping in Tracks	Estimated Wind Speed (m/s)	Median Vulture Altitude (m)	Vulture Altitude Range (m)	Median Glide Angle (degrees)
20150628-A	Laramie, WY	0.890	9.27 + 2	-0.27 + 1.75	3.23	2.7	28 + 23.72	171	-1.71 + 10.61
20150625-E	Laramie, WY	0.897	8.69 + 1.68	0.32 + 1.75	5.72	1.9	35 + 31.13	221	2.1 + 11.76
20150623-A	Laramie, WY	0.898	10.03 + 2.08	0.23 + 2.27	11.15	5.8	18 + 10.38	71	1.36 + 13.31
20150629-A	Laramie, WY	0.899	7.99 + 1.45	-0.75 + 0.9	1.80	2.5	22 + 20.76	111	-5.35 + 6.6
20150701-A	Laramie, WY	0.900	8.27 + 1.7	-0.35 + 1.42	1.22	1.2	49 + 35.58	176	-2.4 + 9.49
20150701b-D	Laramie, WY	0.900	10.48 + 1.69	-0.28 + 1.75	0.84	7.9	23 + 16.31	126	-1.54 + 9.72
20150701b-F	Laramie, WY	0.900	10.35 + 1.7	-0.13 + 1.65	1.04	7.2	32 + 22.24	141	-0.73 + 9.27
20150702-C	Laramie, WY	0.900	10.98 + 2.19	-0.94 + 1.88	0.34	2.7	68 + 50.41	151	-4.76 + 9.88
20150702-E	Laramie, WY	0.900	10.02 + 1.82	-0.29 + 1.66	5.69	2.5	55 + 40.03	221	-1.68 + 9.27
20150702-G	Laramie, WY	0.900	10.23 + 2.85	-0.22 + 1.88	3.46	1.3	37 + 31.13	154	-1.27 + 10.61
20150626-D	Laramie, WY	0.910	9.1 + 1.99	-0.2 + 1.44	0.55	2.6	36 + 31.13	97	-1.32 + 9.07
20160906-A	Alcova, WY	0.967	9.03 + 1.48	-0.69 + 0.74	2.19	1.6	38 + 28.17	296	-4.41 + 4.76
20160906-B	Alcova, WY	0.967	8.88 + 1.23	-0.53 + 0.74	0.99	1.4	36 + 26.69	133	-3.37 + 4.76
20160909-A	Alcova, WY	1.015	10.92 + 1.62	-0.32 + 1.16	5.88	1.1	88 + 54.86	214	-1.66 + 6.11
20160912-A	Alcova, WY	1.036	12.18 + 2.39	0.3 + 1.93	2.40	9.7	24 + 17.79	113	1.39 + 9.45
20150609-A	Chapel Hill, NC	1.130	10.23 + 1.76	0.45 + 1.28	0.00	3.9	104 + 48.93	228	2.56 + 7.48
20150609-B	Chapel Hill, NC	1.130	9.39 + 1.9	-0.1 + 1.2	0.65	2.6	70 + 35.58	287	-0.6 + 7.27

Journal of Experimental Biology • Supplementary information

20150609-C	Chapel Hill, NC	1.130	10.04 + 1.51	-0.67 + 0.99	0.72	3.7	54 + 54.86	276	-3.85 + 5.63
20150612-B	Chapel Hill, NC	1.130	9.98 + 1.79	-0.38 + 1.3	0.32	3.9	66 + 53.37	295	-2.25 + 7.59
20150610-B	Chapel Hill, NC	1.140	9.52 + 1.62	-0.34 + 1.14	0.00	1.7	70 + 63.75	281	-2.08 + 6.7
20150610-C	Chapel Hill, NC	1.140	8.95 + 1.6	0.15 + 1.19	4.23	1.8	59 + 31.13	190	0.95 + 7.78
20150602-A	Chapel Hill, NC	1.150	7.92 + 1.17	-0.33 + 0.93	1.51	2.8	28 + 22.24	148	-2.4 + 6.74
20150605-A	Chapel Hill, NC	1.160	9.8 + 2.49	0.15 + 1.39	2.76	2.7	63 + 53.37	301	0.86 + 8.29
20150605-B	Chapel Hill, NC	1.160	10.22 + 1.62	-0.75 + 0.95	1.51	3.3	73 + 65.23	223	-4.13 + 5.15
20150527-A	Chapel Hill, NC	1.186	9.79 + 1.82	-0.27 + 1.3	0.24	2.5	51 + 34.1	231	-1.59 + 7.54
20150527-B	Chapel Hill, NC	1.186	9.43 + 1.79	-0.44 + 1.08	4.53	2	26 + 13.34	133	-2.7 + 6.65
20150529-A	Chapel Hill, NC	1.227	10.93 + 1.56	-0.48 + 1.35	1.65	4.9	42 + 32.62	174	-2.58 + 7.14
20150529-B	Chapel Hill, NC	1.227	10.14 + 1.66	-0.48 + 1.05	0.14	3.5	50 + 38.55	266	-2.74 + 5.92
20150529-C	Chapel Hill, NC	1.227	10.72 + 1.94	-0.47 + 1.01	0.20	5.2	36 + 28.17	135	-2.6 + 5.52

Journal of Experimental Biology • Supplementary information
